# Longitudinal associations of traditional and cyberbullying victimization with perceived stress in adolescents: findings from the STARS cohort

**DOI:** 10.1186/s12889-026-26395-4

**Published:** 2026-01-29

**Authors:** Sofia Panteli, Peter Friberg, Linda Beckman, Yun Chen

**Affiliations:** 1 School of Public Health and Community Medicine, Institute of Medicine, Sahlgrenska Academy, University of Gothenburg, Box 469, Gothenburg, SE 40530 Sweden; 2 Department of Public Health, Institute of Health Science, Karlstad University, Karlstad, Sweden

**Keywords:** Longitudinal study, Adolescents, Traditional bullying, Cyberbullying, Mental health problems, Sex differences

## Abstract

**Background:**

Perceived stress in adolescence is a known early marker for later mental health problems, including depression. Yet, its links to traditional and cyberbullying remain understudied, especially in longitudinal research. Gender differences in stress may reflect varying bullying experiences, but this connection is still unclear. This longitudinal study investigated whether victimization by traditional bullying and/or cyberbullying between ages 13 and 15 is associated with increased self-perceived stress, and whether these associations differ by sex or the form of victimization.

**Methods:**

We used data from the baseline (age 13) and two-year follow-up (age 15) surveys of the STARS (Study of Resilience and Stress) cohort in Sweden. The sample included 2099 adolescents (44% male and 56% female). Perceived stress was measured by Cohen’s Perceived Stress Scale, while traditional bullying and cyberbullying victimization were each assessed using a single-item question. Statistical analyses included t-tests, chi-square tests, one-way ANOVAs, and General Linear Models for repeated measures adjusting for confounders in the whole sample, and sex-stratified analyses.

**Results:**

Perceived stress increased between ages 13 and 15 for both sexes, with females consistently reporting higher stress levels than males. The prevalence of traditional bullying and cyberbullying victimization rates ranged from 8.2–11.2% and 7.1–9.9%, respectively, and generally declined over the two years. Females were more exposed to cyberbullying victimization at both time points (8.3–9.9%) compared to males (5.3–5.5%). In both sexes, experiencing bullying–whether traditional, cyber, or both–at the age 13, 15 or at both time points was associated with increased stress levels over the two years. The impact on stress persisted even without continued victimization. Although overall patterns were similar between males and females, being cyberbullied at age 15 was specifically associated with increased stress among females, but not males. Changes in stress levels were similar across both forms of victimization.

**Conclusions:**

Exposure to traditional and/or cyberbullying at age 13, 15, or across both time points is associated with increased perceived stress over time in both males and females, highlighting the potentially lasting impact of victimization on adolescent stress trajectories and the importance of preventive and supportive interventions.

**Supplementary Information:**

The online version contains supplementary material available at 10.1186/s12889-026-26395-4.

## Background

Over one in ten individuals aged 5–24 worldwide live with at least one diagnosed mental disorder [1]. In Sweden, the proportion of 13- and 15-year-old pupils reporting psychosomatic symptoms continues to increase, reaching the highest level since the mid-1980s, peaking in 2021–22 [2]. This upward trend is concerning, as such symptoms during adolescence have been shown to predict depression and anxiety symptoms in young adulthood [3–5]. Self-reported stress accounts for 49% of the variation in adolescents’ psychosomatic symptoms, making it a major contributing factor [6]. Among stressors, bullying victimization stands out as particularly significant, especially during early and mid-adolescence, a developmental stage marked by increased peer influence [7].

Growing evidence links both traditional bullying and cyberbullying—most common in early to mid-adolescence—to poor mental health outcomes, including suicidal thoughts, depression, anxiety, and behavioural or social problems [8–11]. Perceived stress is increasingly recognized as an early marker for severe mental health problems; for example, a prospective study found that, relative to low stress, moderate stress at age 16 doubled the odds of developing a mental disorder within a year, and high stress increased the odds sixfold [12]. Despite growing awareness, few studies have specifically examined the relationship between stress and bullying during early to mid-adolescence, particularly using longitudinal designs capable of tracking change and associations over time.

Recent studies have highlighted the prevalence of bullying and digital device use among youth. The Global School-based Student Health Survey found that 8.4% of European adolescents (ages 12–17) experienced traditional bullying [13]. A Norwegian study showed that 13.4% of adolescents (ages 13–16) reported traditional bullying, while 11.9% were cyberbullied in the past six months [14]. As digital communication becomes an increasingly central part of adolescents’ daily lives, opportunities for online interactions—and consequently, exposure to cyberbullying—have grown. Accordingly, digital engagement among youth has increased substantially, with 38% of primary and 69% of secondary school students using electronic devices for two hours or more daily [15]. These findings emphasize the dual challenges of peer victimization and increased screen time among youth. Social media, especially anonymous platforms, is a key venue for cyberbullying [16]. Adolescents who use social networking sites are found to experience higher levels of cyberbullying than non-users [17].

Cross-sectional studies consistently show that adolescents exposed to bullying report higher psychological distress and perceived stress compared with their non-victimized peers [12, 18–20]. There is also emerging mechanistic evidence: among early adolescents, peer victimization has been associated with changes in cortisol reactivity [19, 21, 22], which in turn relates to heightened depressive symptoms [21]. Given the widespread use of electronic devices among youth [15], exposure to cyberbullying—and its attendant stressors—is likely to be pervasive. Importantly, cyberbullying differs from traditional bullying in ways that can intensify perceived stress: the criterion of power imbalance central to traditional definitions is less clearly specified online, and a single harmful act can be rapidly reshared and viewed by large audiences, creating a networked power asymmetry while undermining the requirement of intentional repetition that underpins traditional bullying [23].

A recent meta-analysis examining traditional and cyberbullying found substantial overlap—approximately two-thirds of cyberbullying victims also experienced traditional bullying—and the combined exposure conferred the highest risk of mental health problems [24]. Although cyberbullying victims showed slightly higher risks of self-harm and suicidal ideations or attempts than those traditionally bullied, this difference was not statistically significant. Importantly, most studies in the meta-analysis were cross-sectional. This underscores a clear need for longitudinal research beginning in early adolescence to delineate trajectories of perceived stress and the unique versus shared effects of the two bullying forms. Consistent with transactional models, the limited longitudinal evidence suggests that psychological distress and peer victimization can influence one another over time [25, 26]; in some studies, distress (including perceived stress) predicts subsequent victimization more strongly than the reverse [26]. Another study showed that cyberbullying victimization predicted perceived stress four months later [22].

Emerging evidence also points to notable sex differences in these experiences. More girls than boys reported cyberbullying victimization [14], and are more likely to experience depression afterwards, possibly due to different online behaviours [27]. Among females, the likelihood of mental health problems increases with the frequency of being cyberbullied, a trend not observed among males [8]. Teenage girls also report higher stress levels than boys [6, 28]. However, it remains unclear whether these differences in perceived stress are directly linked to variations in bullying experiences.

This longitudinal study aimed to determine whether traditional and/or cyberbullying victimization from early to mid-adolescence is associated with increased perceived stress. It had three objectives: 1) to examine changes in stress levels and bullying prevalence from ages 13 to 15; 2) to investigate whether exposure to one or both forms of bullying was associated with changes in stress; 3) to explore whether these patterns differ by sex or type of victimization.

## Methods

### Study design, setting, and participants

A longitudinal study design was used. Participants were seventh-grade pupils recruited for the Study of Adolescence Resilience and Stress (STARS), an ongoing longitudinal study with baseline examinations and three follow-ups (2-year, 5-year, and a planned 10-year follow-up). Participants were recruited from 54 schools across 16 municipalities in Western Sweden during 2015–2019. Schools were intentionally selected to represent a wide range of socioeconomic contexts. With approval from the school principals, members of the research team visited 7th-grade classes to inform pupils and their teachers about the study. Information letters were distributed to pupils and their parents or guardians. In total, 5,084 pupils were informed about the study and invited to participate. After obtaining written informed consent from both pupils and their parents (or guardians), examinations were scheduled and conducted at the pupils’ respective schools, where they completed an online questionnaire and underwent physical assessments.

The study was conducted in accordance with the principles outlined in the Declaration of Helsinki. Ethical approval was obtained from the Swedish Ethical Review Authority. Written informed consent was obtained from both participants and their parents or guardians for the use of data in research. At baseline, 2,283 individuals participated, and 2,152 took part in the two-year follow-up. For the analyses presented in this study, participants with missing data at either baseline or the two-year follow-up were excluded, resulting in a final sample size of 2,099. Some differences between participants included in the analyses and those excluded were observed; details are provided in Table S1 of the supplementary material.

### Traditional bullying and cyberbullying victimization

Traditional bullying victimization was assessed using the general question: “Have you been bullied in the last few months?”, adapted from the Bully/Victim Questionnaire [29]. This question was preceded by the definition of traditional bullying as used in the World Health Organization Health Behavior in School-Aged Children study [2]: “*A person is being bullied when others say or do mean and unpleasant things to him or her. It is also bullying when someone is constantly teased in a way that he or she does not like. But it is not bullying when two fairly equally strong individuals argue or fight*”.

Cyberbullying victimization was measured using the general question: “Have you been cyberbullied in the last few months?”, adapted from Slonje and Smith [30, 31]. This question was preceded by a definition of cyberbullying adapted from Frisén et al. [32]: “*A person is being cyberbullied when he or she is repeatedly subjected to harmful or abusive behaviour by one or more individuals *via* the internet and mobile phone. It is difficult for the person to defend themselves*”.

For both questions, five response options were given: “no”, “yes once or twice”, “yes 2 or 3 times per month”, “yes, about once a week”, and “yes, several times per week”. The five ordinal responses were categorized into a binary variable for both traditional and cyberbullying victimization, where 0 = “never” and 1 = ”once or more”. Different cut-off levels have been used in earlier research [7, 33]. The chosen cut-off level reflects the theoretical discussion on whether repetition is a necessary criterion for cyberbullying [23]. In addition, our empirical observations supported this threshold, as participants who reported being bullied “once or twice” already showed elevated levels of self-perceived stress compared with those who reported no bullying (data not shown). Additionally, this cut-off level has been used in previous studies [31, 34].

To capture patterns of traditional bullying victimization over time, a four-level categorical variable was created based on responses at baseline and the two-year follow-up. Participants were classified as follows: “No” if they reported no bullying at either time point; “T1” if they reported being bullied only at baseline; “T2” if they reported being bullied only at two-year follow-up; and “T1&T2” if they reported being bullied at both time points. An identical four-level variable was constructed for cyberbullying victimization using the same criteria. In the following text, these groups are referred to as *No*, *T1*, *T2*, and *T1&T2*.

### Perceived stress

Perceived stress was measured using the 10-item Perceived Stress Scale [35]. It is a well-validated and widely used scale in both young people and adults. The items measure the extent to which respondents perceive their lives as unpredictable, uncontrollable, and overloading. An example item is: “In the last month, how often have you felt that you were unable to control the important things in your life?”. Participants rated each statement on a 5-point scale from 0 (never) to 4 (very often). A maximum of one missing item was allowed; in such cases, the missing item was replaced by the respondent’s mean score across the completed items. The total score, based on 10 items, ranged from 0 to 40, with higher scores representing higher levels of perceived stress. Cronbach’s alpha coefficient was 0.815 at baseline and 0.774 at two-year follow-up, indicating high internal consistency reliability of the scale.

### Potential confounders

Sex at birth, immigrant background, and parents’ education were included as confounders in the analysis because previous research has shown that they are related to both stress and bullying [6, 36]. Sex at birth was derived from the adolescent’s social security number, which contains a specific digit identifying sex assigned at birth. Data on parental education levels were obtained from Statistics Sweden through data linkage using the adolescent’s social security number. Parents’ education level was categorized based on the highest educational level attained by either parent: primary, secondary, or postsecondary. Immigrant background was derived from three questions about the birth countries of the adolescent and both parents. Adolescents born in Sweden with at least one Swedish-born parent were classified as having a Swedish background; those born abroad or with both parents born abroad were classified as having an immigrant background.

### Statistical analysis

Chi-square tests were used to compare categorical variables across groups. For continuous variables, Student’s t-tests were conducted to analyse differences between two groups, while one-way ANOVA followed by Bonferroni correction was used to assess differences among three or more groups. The assumption of normality was satisfied after the visual inspection of histograms and normal probability quantile plots (Q-Q plots).

General Linear Models (GLM) for repeated measures were used to assess group differences over time. Time was specified as the within-subjects factor, while traditional bullying or cyberbullying victimization groups served as the between-subject factors. In the analyses involving the whole sample, sex, parents’ education, and migrant background were included as confounders. In the sex-stratified analyses, parents’ education and migrant background were included as confounders. A total of 136 individuals reported experiencing both traditional bullying and cyberbullying victimization. Additional GLM analyses were conducted, excluding these individuals to isolate the distinct effects of each form of victimization. All analyses were conducted using Statistical Package for the Social Sciences (SPSS) version 30. P ≤ 0.05 was considered statistically significant.

## Results

### Changes over the two years and sex-related differences

Over the two years, the prevalence of being traditionally bullied declined from 11.0% at age 13 to 8.2% at age 15; similar decreases were seen in males (10.7% to 7.1%) and females (11.2% to 9.1%) (Table 1). The prevalence of cyberbullying victimization also decreased slightly, 7.9% at age 13 and 7.1% at age 15, except among males, where the prevalence of cyberbullying victimization remained relatively stable (5.3% to 5.5%). Over the same period, perceived stress levels increased (Table 1).

**Table 1:** Descriptive statistics of the study variables in the total sample, as well as males and females separately

	Total N (%)	Males N (%)	Females N (%)	χ^2^, p
Immigrant background				
Immigrant	383 (18.2)	180 (19.5)	203 (17.3)	
Swedish	1,716 (81.8)	744 (80.5)	972 (82.7)	0.194
Parents’ education				
Primary school	71 (3.4)	29 (3.1)	42 (3.6)	
Secondary school	554 (26.4)	232 (25.1)	322 (27.4)	
Post-secondary school	1,474 (70.2)	663 (71.8)	811 (69.0)	0.392
**Baseline**				
Traditionally bullied				
No	1,868 (89.0)	825 (89.3)	1,043 (88.8)	
Yes	231 (11.0)	99 (10.7)	132 (11.2)	0.706
Cyberbullied				
No	1,934 (92.1)	875 (94.7)	1,059 (90.1)	
Yes	165 (7.9)	49 (5.3)	116 (9.9)	< 0.001
**2-year follow-up**				
Traditionally bullied				
No	1,926 (91.8)	858 (92.9)	1,068 (90.9)	
Yes	173 (8.2)	66 (7.1)	107 (9.1)	0.104
Cyberbullied				
No	1,951 (92.9)	873 (94.5)	1,078 (91.7)	
Yes	148 (7.1)	51 (5.5)	97 (8.3)	0.015
	Mean (95% CI)	Mean (95% CI)	Mean (95% CI)	t-test, p
Baseline				
Age	13.5 (13.5–13.6)	13.5 (13.5–13.6)	13.6 (13.6–13.6)	0.058
Self-perceived stress	15.5 (15.2–15.7)	13.7 (13.4–14.1)	16.8 (16.5–17.2)	< 0.001
2-year follow-up				
Age	15.5 (15.5–15.5)	15.5 (15.5–15.5)	15.5 (15.5–15.5)	0.166
Self-perceived stress	17.0 (16.7–17.2)	14.5 (14.1–14.9)	18.9 (18.5–19.3)	< 0.001

*CI* Confidence interval

Sex difference was observed in cyberbullying victimization: 5.3% males and 9.9% females at age 13; 5.5% males and 8.3% females at age 15. Furthermore, females reported higher levels of stress compared to males, and the sex-related gap in perceived stress widened at age 15 (Table 1).

### Stress trajectories following bullying exposure: differences by sex and type of victimization

Compared with the *No* group, higher stress was observed at both time points among the *T1*, *T2*, and *T1&T2* traditional bullying victimization groups (Table 2); the same pattern held for cyberbullying victimization (Table 3). Sex, immigrant background, and parental education also differed across groups (Tables 2 & 3).

**Table 2:** Descriptive statistics of adolescents and their levels of perceived stress in traditional bullying victimization groups

Traditional bullying victimization groups	No N (%)	T1 N (%)	T2 N (%)	T1&T2 N (%)	χ^2^, p
Males	785 (44.6)	73 (43.7)	40 (36.7)	26 (40.6)	
Females	974 (55.4)	94 (56.3)	69 (63.3)	38 (59.4)	0.401
Immigrant background					
Immigrant	304 (17.3)	50 (29.9)	13 (11.9)	16 (25.0)	
Swedish	1,455 (82.7)	117 (70.1)	96 (88.1)	48 (75.0)	< 0.001
Parents’ education					
Primary school	57 (3.2)	5 (3)	4 (3.7)	5 (7.8)	
Secondary school	455 (25.9)	42 (25.1)	38 (34.9)	19 (29.7)	
Post-secondary school	1,247 (70.9)	120 (71.9)	67 (61.5)	40 (62.5)	0.156
Self-perceived stress	Mean (95%CI)	Mean (95%CI)	Mean (95%CI)	Mean (95%CI)	t-test, p
T1 (Age 13)	14.8 (14.5–15.1)	19.6^a ^(18.7–20.5)	16.8 (15.6–17.7)	20.8^a ^(19.2–22.3)	< 0.001
T2 (Age 15)	16.4 (16.1–16.7)	18.2 (17.3–19.2)	20.7^a^ (19.6–21.8)	21.5^a ^(19.9–23.0)	< 0.001

*CI* Confidence interval

^a^indicates that values in the same row did not differ significantly from each other

**Table 3:** Descriptive statistics of adolescents and their perceived stress levels in cyberbullying victimization groups

Cyberbullying victimization groups	No N (%)	T1 N (%)	T2 N (%)	T1&T2 N (%)	χ^2^, p
Males	835 (45.6)	38 (31.4)	40 (38.5)	11 (25)	
Females	995 (54.4)	83 (68.6)	64 (61.5)	33 (75)	< 0.001
Immigrant background					
Immigrant	321 (17.5)	32 (26.4)	18 (17.3)	12 (27.3)	
Swedish	1,509 (82.5)	89 (73.6)	86 (82.7)	32 (72.7)	0.036
Parents’ education					
Primary school	63 (3.4)	5 (4.1)	2 (1.9)	1 (2.3)	
Secondary school	462 (25.2)	37 (30.6)	39 (37.5)	16 (36.4)	
Post-secondary school	1,305 (71.3)	79 (65.3)	63 (60.6)	27 (61.4)	0.065
Self-perceived stress	Mean (95%CI)	Mean (95%CI)	Mean (95%CI)	Mean (95%CI)	t-test, p
T1 (age 13)	14.8 (14.6–15)	21.3^a^(20.1–23)	17.7 (16.6–18.8)	21.8^a^(19.8–23.8)	< 0.001
T2 (age 15)	16.5 (16.2–16.8)	19.1^a^(17.8–20.3)	19.9^a^(18.5–21.3)	22.8 (21–24.6)	< 0.001

*CI* Confidence interval

^a^indicates that values in the same row did not differ significantly from each other

We next examined whether changes in stress levels over the two years varied across different traditionally bullying or cyberbullying victimization groups, using GLM for repeated measures and adjusted for sex (for whole sample analyses), immigrant background, and parents’ education. The GLM for repeated measures revealed a statistically significant interaction between time and victimization groups, indicating group-specific changes in stress over time.

**Fig. 1:**
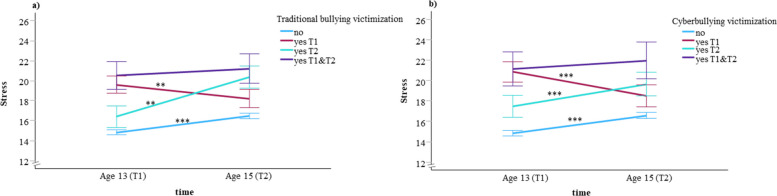
Levels of perceived stress among traditionally bullied (**a**) and cyberbullied (**b**) groups in the total sample. Values are adjusted for sex, parents’ education and immigrant background, and expressed as means with error bars representing 95% confidence intervals. **p < 0.01 vs baseline, ***p < 0.001 vs baseline

Figure 1 illustrates the results for the entire sample. The patterns were largely consistent whether grouped by traditional bullying victimization (Fig. 1a) or cyberbullying victimization (Fig. 1b). Compared to age 13, the stress levels increased considerably at age 15 in both the *No* and *T2* groups, but not in the *T1&T2* groups. Although the *T1* group showed a significant decline over the two years, their stress levels remained considerably higher than those in the *No* group at both time points. Notably, the *T2* group already showed elevated stress at baseline, which rose further at the two-year follow-up. The *T1&T2* group consistently reported the highest stress levels throughout the study period.

**Fig. 2:**
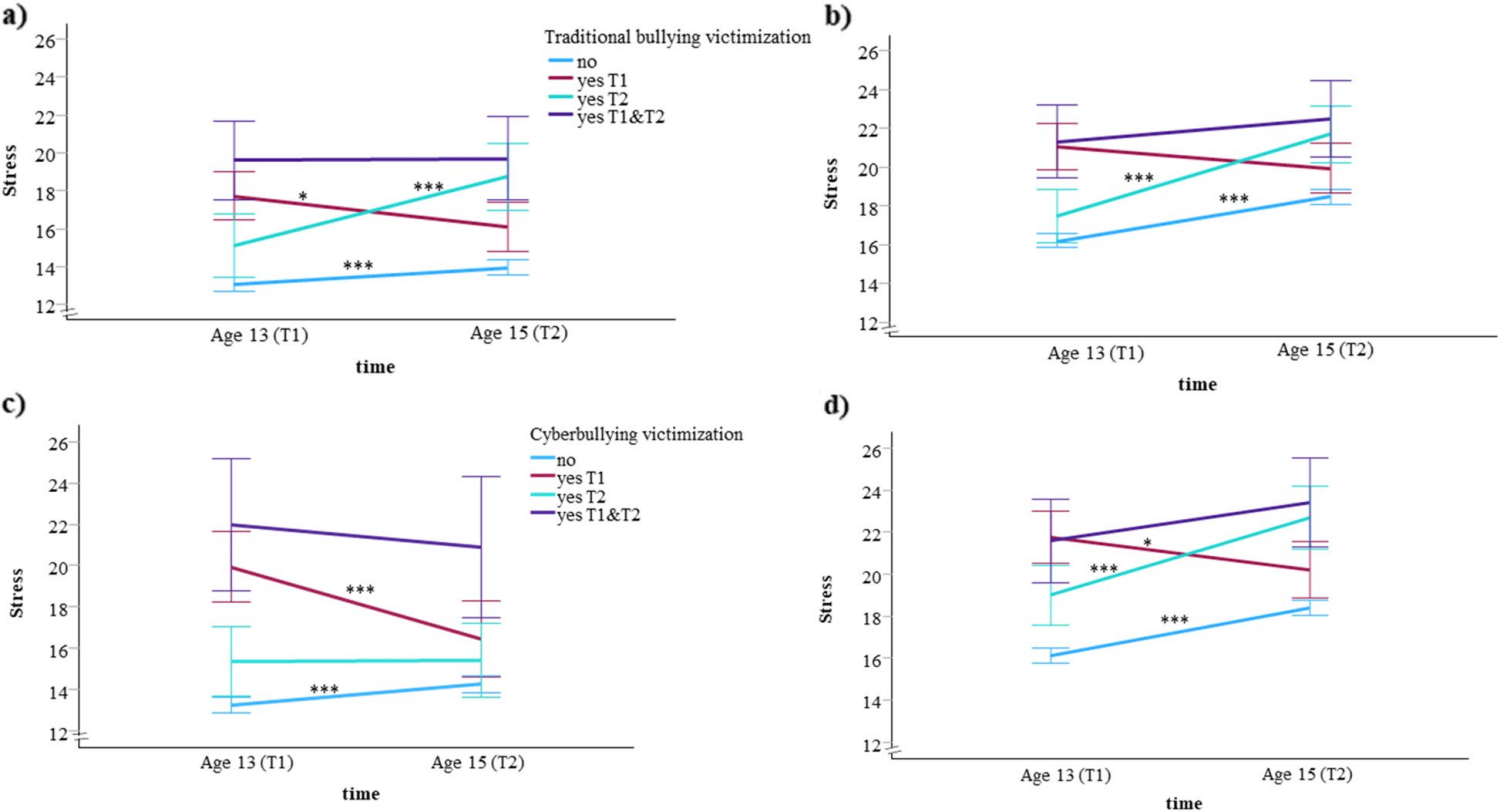
Levels of perceived stress across traditionally bullied (**a**, **b**) and cyberbullied (**c**, **d**) groups in males (**a**, **c**) and females (**b**, **d**). Values are adjusted for parents’ education and immigrant background, and expressed as means with error bars representing 95% confidence intervals. *p < 0.05 vs baseline, ***p < 0.001 vs baseline

A significant interaction between time and sex was also found, suggesting a sex-related difference in stress trajectories. Consequently, GLMs were conducted separately for males and females. In both groups, the interaction between time and traditional bullying or cyberbullying victimization was statistically significant. As illustrated in Fig. 2, the patterns show both similarities and differences between males and females, whether categorized by traditional bullying (Fig. 2a–b) or cyberbullying victimization (Fig. 2c–d).

For traditional bullying victimization, similar stress level patterns were observed in both males (Fig. 2a) and females (Fig. 2b) in the *No* and *T2* groups, with stress levels increasing at age 15. Stress levels in the *T1&T2* group were highest among all four groups, with no significant changes observed between ages 13 and 15 in either males or females. In contrast, the *T1* group showed a decline in stress levels over the study period; however, this reduction was statistically significant only in males. Notably, despite this decrease, stress levels in the *T1* group remained higher than those in the *No* group at age 15. Interestingly, stress levels in the *T2* group were already higher than those in the *No* group at age 13 for both males and females and increased further at age 15.

In the context of cyberbullying victimization, the patterns of change in stress were generally similar to those observed across the traditional bullying victimization groups, except for the *T2* group (Fig. 2c-d). In the *T2* group, stress levels were already elevated at age 13 compared to the *No* group and increased further at age 15 in females (Fig. 2d), but not in males (Fig. 2c). For males, stress levels in the *T2* group did not differ from those in the *No* group.

The stress levels in *T1&T2* remained the highest throughout the study period (Tables 2 and 3) and did not significantly differ between males and females [bullying: (t(62) = −1.017, *p* = 0.313); cyberbullying: (t(42) = 0.197,* p* = 0.845)]. No significant interaction was found between sex, bullying/cyberbullying victimization, and time [(traditional bullying: (F(3,2,069) = 0.601, *p* = 0.614; cyberbullying: (F(3, 2,069) = 0.605, *p* = 0.612)].

Furthermore, excluding 136 individuals who reported experiencing both traditional bullying and cyberbullying victimization did not alter the overall patterns of changes in perceived stress over the two years (see Fig. S1a-S1b in the supplementary material). However, stress levels were generally higher when individuals with dual victimization were included, compared to when they were excluded.

In sensitivity analyses, applying a “2–3 times per month” cutoff did not change the patterns in perceived stress levels or their two-year changes across the four groups (Tables S2 and S3).

## Discussion

Our longitudinal findings enhance the understanding of how early adolescent experiences of traditional bullying and cyberbullying shape stress trajectories over time.

Four key findings emerged from the present study. First, stress levels increased for both sexes between the ages of 13 and 15. Second, the prevalence of traditional bullying and cyberbullying victimization decreased over the two years, except among males, where the prevalence of cyberbullying victimization remained relatively stable. Third, adolescents who experienced bullying at age 13, 15, or both these ages reported higher stress levels than those not bullied, with the highest stress observed in those bullied at both time points. The impact of bullying victimization on stress persisted over time, even without continued exposure. Changes in stress levels were similar across both forms of victimization. Finally, regarding sex differences, females reported more cyberbullying victimization and higher stress levels than males at both time points. While the overall patterns linking bullying victimization to stress were largely similar across sexes, being cyberbullied at age 15 was associated with increased stress among females but not males.

Our results are in line with previous studies, which found that adolescents exposed to traditional bullying [19] or cyberbullying [20] were more likely to report higher stress levels than non-bullied students. Similarly, it has been reported that 15-year-old victims of cyberbullying experienced higher stress levels than cyberbystanders (those who observed but were not directly involved in a cyberbullying incident) [22]. Taken together, these findings suggest that repeated exposure to bullying may have a cumulative effect on adolescents’ stress levels, emphasizing the importance of early intervention and continuous support from both parents and school staff.

Importantly, our study revealed a clear sex difference in the context of cyberbullying. Being cyberbullied at age 15 was associated with increased stress among females, but not males. This finding aligns with previous research showing that even a single instance of cyberbullying significantly increased the risk of psychological distress in females, but not in males [8]. This disparity has been attributed to gendered socialization processes, wherein girls are often encouraged to be more emotionally attuned and sensitive to others’ opinions [27]. The differences may also stem from various digital habits between males and females. For instance, a study of Norwegian adolescents aged 11–19 found that boys spent more time playing video games on consoles or computers, while girls spent more time on social media [37]. These differing patterns of digital engagement may contribute to females’ heightened vulnerability to cyberbullying and its psychological consequences. Supporting this, females who were bullied in grades 9 and 11 were more likely to have depressive and anxiety symptoms at ages 20–21 [38]. Similarly, a prospective study found that being bullied at school between ages 10 and 18 predicted psychological complaints ten years later in women, but not in men [11]. Another factor that may contribute to the differential impact of cyberbullying is the digital context in which it occurs. One might surmise that being targeted on a social media platform due to the posting of a photo may have a different psychological impact compared to being cyberbullied on a gaming platform for poor performance. Future research should investigate the specific digital contexts in which cyberbullying occurs and how they differentially affect stress levels in males and females.

Interestingly, we did observe that males reported elevated stress when cyberbullied at age 13. This suggests that the age at which cyberbullying occurs may play an important role in the appraisal of the incident in males. Developmental changes between ages 13 and 15 may result in more effective coping mechanisms in response to cyberbullying in males at age 15. Future studies should examine sex-specific coping strategies to better understand these variations.

Our findings are consistent with some previous studies on sex-specific effects of bullying, while they diverge from others. Exposure to bullying during adolescence was cross-sectionally linked with anxiety and depressive symptoms in both sexes, but only females showed long-term effects into adulthood [11]. Similarly, a long-term effect of bullying victimization on mental health has been found in girls, but not in boys, despite the follow-up occurring two years later in 14-year-olds [39]. These differences may partly be due to differences in outcome measures, as prior studies focused on general psychological symptoms, whereas we assessed perceived stress specifically. This distinction highlights the need for future research to explore sex differences in perceived stress as a unique dimension of bullying-related outcomes.

In our study, we observed that although the stress levels in the *T1* group declined somewhat over the two years, they remained significantly higher than those in the *No* group. Thus, experiencing bullying—whether traditional, cyber, or both—at a single time point around age 13 can have a long-lasting impact on stress levels, persisting even two years after the incident. Previous research has argued that even brief episodes of cyberbullying may lead to severe psychological effects, partly due to the potentially wide audience reached through digital platforms [30].

Intriguingly, adolescents in the *T2* group reported higher stress levels than the *No* group not only at age 15 but also at age 13, despite not having experienced bullying at age 13. The current study does not provide a definitive explanation for this observation. One possibility is that adolescents in the *T2* group may possess distinct psychological characteristics, such as heightened stress sensitivity, which could predispose them to perceive everyday situations as more stressful. These elevated baseline stress levels might, in turn, predict later victimization. Supporting this notion, a previous study found that boys often described using bullying to release their aggression, typically targeting peers perceived as “weaker” [40]. Furthermore, research has highlighted that adolescents who experience bullying often report lower levels of social support from both peers and adults [11]. This lack of support may contribute to the power imbalance that defines bullying and could also explain the elevated stress levels observed in the *T2* group at age 13, before they experienced being bullied at age 15. These findings suggest that being anxious and sensitive to stressful events may elevate the risk of subsequent bullying victimization. Consistent with this, earlier work shows that psychological distress (including perceived stress) prospectively predicts later victimization [25, 26].

In the present study, the prevalence of traditional bullying and cyberbullying victimization ranged from 7.1–11.2% and 5.3–9.9%, respectively, between early and mid-adolescence. In comparison, a cross-sectional study of adolescents aged 13–15 using the same cut-off criteria reported lower overall rates of traditional (4.9% of males; 5.0% of females) and cyberbullying victimization (1.5% of males; 3.2% of females) [34]. The higher prevalence of cyberbullying victimization observed in our study may be explained by the increasing accessibility and use of digital technologies among adolescents in recent years. For instance, by 2018, more than 95% of adolescents aged 13–16 globally owned a smartphone [41], which may have heightened their exposure to online interactions and, consequently, cyberbullying.

We found no significant sex differences in traditional bullying victimization, consistent with the findings by Beckman et al. [34]. However, this contrasts with another study on mid-adolescents, which reported notably higher rates among boys [19]. On the other hand, our results did show that females experienced more cyberbullying than males at both baseline and the two-year follow-up. These results align more closely with those reported by Låftman et al. [38], who reported slightly higher overall bullying rates among females aged 15–18, though their study did not distinguish between traditional and cyberbullying, limiting comparability. Similarly, Kyrrestad et al. found no significant gender differences in traditional bullying victimization among adolescents aged 12–16 but did report higher rates of cyberbullying victimization among females (15.1%) than males (8.7%) [14].

We observed clear sex differences in perceived stress, with females reporting higher stress levels than males at both time points. This finding aligns with previous research showing that girls generally reported higher levels of perceived stress compared to boys [6, 28]. These differences may originate from variations in how males and females appraise and respond to stressful life events, as well as differences in their propensity to report stress and mental health concerns. Girls are more likely to internalize challenges and demands, often setting higher personal aspirations and expressing greater concern about the future [28]. These tendencies can contribute to elevated and cumulative stress levels over time [28].

The present study also found that adolescents who were not bullied had higher stress levels at the two-year follow-up compared to baseline. This suggests that factors other than bullying, such as increased school workload or stressful events within the family, influence stress levels in adolescents.

### Strengths and limitations

A key strength of this study is its longitudinal design, which tracks development from early to mid-adolescence. However, we did not explicitly measure the possible bidirectional links between stress and victimization. Although elevated stress levels in the *T2* group at age 13, before reported bullying at age 15, are consistent with the idea that stress and mental health problems may increase vulnerability to later victimization, this interpretation should be made cautiously. Moreover, the cutoff used to identify those being bullied may limit comparability with studies using different thresholds or measures. Bullying and cyberbullying were assessed using single-item measures. Bullying and stress were assessed through self-report, which may be subject to reporting bias. In addition, the measure did not capture the severity or chronicity of bullying, which could be relevant to its association with stress. Future research using more comprehensive, continuous, and multi-item measures is needed to examine these issues in greater depth.

Despite a very high follow-up rate (95%), non-response analysis revealed that excluded individuals were more likely to have an immigrant background, lower parental education, and higher baseline levels of bullying and stress. These differences suggest that the association between bullying victimization and perceived stress may have been underestimated. As only sex assigned at birth was recorded, potential influences of gender identity could not be examined.

## Conclusion

The present study demonstrates that adolescents who experienced bullying at age 13, 15, or both reported significantly higher stress levels, with persistent effects even without ongoing victimization. Moreover, females experienced more cyberbullying and higher stress than males and being cyberbullied at age 15 was linked to increased stress over the following 2 years in females, but not males. These findings underscore the enduring psychological impact of bullying and cyberbullying during early adolescence and highlight the importance of early intervention strategies.

## Supplementary Information

Supplementary Material 1.

## Data Availability

The datasets generated and analyzed in this study are not publicly accessible due to the inclusion of sensitive personal information. Specifically, the STARS study involves personal data, and the linked register data from Statistics Sweden also contain identifiable information. As such, access to these datasets is restricted under the Swedish Public Access to Information and Secrecy Act. However, researchers interested in collaborating on the STARS study are encouraged to contact Yun Chen (yun.chen@gu.se) for further information.
