# Supplementary material for: Longitudinal associations of traditional and cyberbullying victimization with perceived stress in adolescents: findings from the STARS cohort
Source: BMC Public Health. 2026 Jan 29;26:698. doi: 10.1186/s12889-026-26395-4 (PMC12924448; doi:10.1186/s12889-026-26395-4)

| **Table S1.** Descriptives statistics of study variables at baseline in excluded and included cases and χ^2^ or t-test for group comparisons | | | |
| --- | --- | --- | --- |
|  | **Included**  (n=2,099) | **Excluded**  (n=184) | **χ^2^, p** |
| Males  Females | 44.0%  56.0% | 49.5%  50.5% | 0.155 |
| Ethnic background |  |  |  |
| Immigrant | 18.2% | 54.3% |  |
| Swedish | 81.8% | 45.7% | <0.001 |
| Parents’ education |  |  |  |
| Primary school | 3.4% | 14.4% |  |
| Secondary school | 26.4% | 35.6% |  |
| Post-secondary school | 70.2% | 50.0% | <0.001 |
| Traditional bullying victimization  No  Yes | 89.0%  11.0% | 82.7%  17.3% | 0.011 |
| Cyberbullying victimization  No  Yes | 92.1%  7.9% | 86.1%  13.9% | 0.005 |
|  | **Mean (95%CI)** | **Mean (95%CI)** | **t-test, p** |
| Age | 13.6 (13.5-13.6) | 13.7 (13.6-13.7) | 0.002 |
| Self-perceived stress | 15.5 (15.2-15.7) | 16.7 (15.8-17.8) | 0.006 |

CI: Confidence interval

| **Table S2.** Perceived stress, expressed as Mean (95% confidence interval), in traditional bullying victimization groups identified using the cutoff “2 or 3 times per month”. | | | | | | |
| --- | --- | --- | --- | --- | --- | --- |
| **Traditional bullying**  **victimization groups** | **No**  **(N=1974)** | **T1**  **(N=67)** | **T2**  **(N=45)** | **T1&T2**  **(N=13)** | **ANOVA p** |  |
| Baseline (T1) | 15.2  (14.9-15.5) | 20.1^a^  (18.4-21.8) | 17.3  (15.6-19.1) | 23.5^a^  (18.8-28.2) | <0.001 |  |
| 2-year follow-up (T2) | 16.7  (16.4-17) | 19.6^a^  (18-21) | 22.4^a^  (21-24) | 23.4^a^  (19-27.6) | <0.001 |  |

^a^indicates that values in the same row did not differ significantly from each other, *p* < .001.

| **Table S3.** Perceived stress, expressed as Mean (95% confidence interval), in cyberbullying victimization groups identified using the cutoff “2 or 3 times per month”. | | | | | |
| --- | --- | --- | --- | --- | --- |
| **Cyberbullying victimization groups** | **No**  **(N=2023)** | **T1**  **(N=44)** | **T2**  **(N=25)** | **T1&T2**  **(N=7)** | **ANOVA p** |
| Baseline (T1) | 15.2  (15-15.4) | 23.5^a^  (21.4-25.6) | 16.2  (13.6-18.8) | 28.1^a^  (21.4-34.8) | <0.001 |
| 2-year follow-up (T2) | 16.8  (16.5-17.1) | 20.5^a^  (18.4-22.7) | 19.3^a^  (15.6-22.6) | 27^a^  (20.4-33.6) | <0.001 |

^a^indicates that values in the same row did not differ significantly from each other, *p* < .001.

**Figure S1**

Levels of perceived stress across only traditionally bullied (a) and only cyberbullied (b) group in the total sample, excluding individuals who experienced both forms of victimization.

Values are adjusted for sex, parental education and immigrant background, and expressed as means with error bars showing 95% confidence intervals. *p < .05, ***p < .001 vs T1.


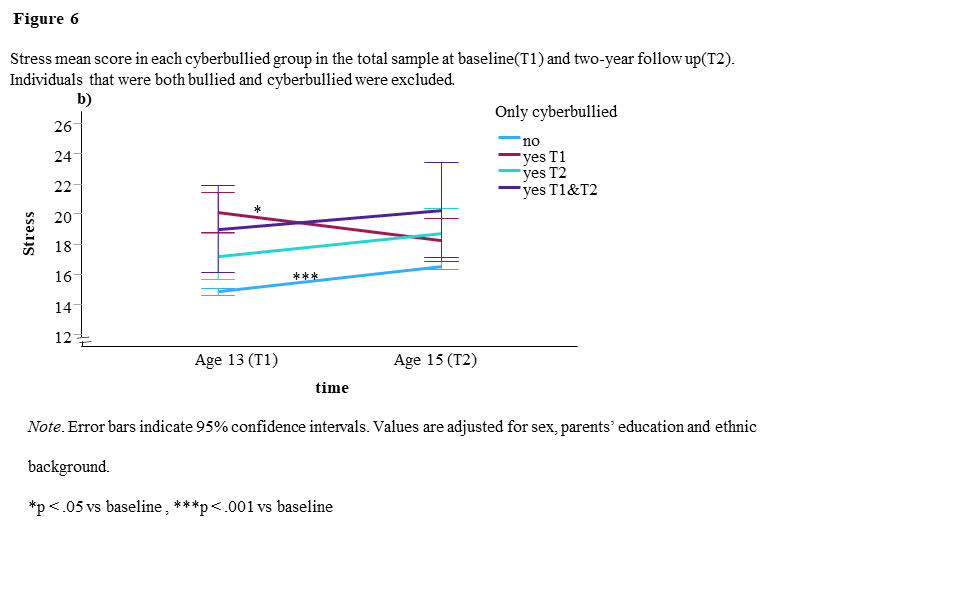

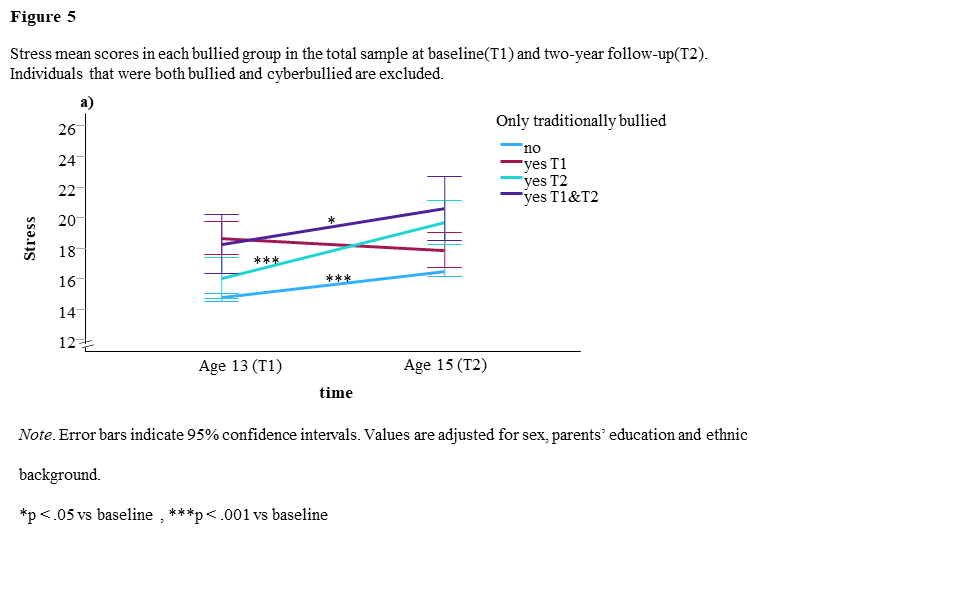

Supplement: Supplementary file 1 — Supplementary Material 1. [file 12889_2026_26395_MOESM1_ESM.docx]
